# Histopathological findings in a COVID-19 patient affected by ischemic gangrenous cholecystitis

**DOI:** 10.1186/s13017-020-00320-5

**Published:** 2020-07-02

**Authors:** Andrea Bruni, Eugenio Garofalo, Valeria Zuccalà, Giuseppe Currò, Carlo Torti, Giuseppe Navarra, Giovambattista De Sarro, Paolo Navalesi, Federico Longhini, Michele Ammendola

**Affiliations:** 1 Anesthesia and Intensive Care, Department of Medical and Surgical Sciences, “Magna Graecia” University, Catanzaro, Italy; 2 Pathology Unit, “Pugliese-Ciaccio” Hospital, Viale Pio X°, 88100 Catanzaro, Italy; 3 Digestive Surgery Unit, Department of Science of Health, “Magna Graecia” University, Catanzaro, Italy; 4 Surgical Oncology Division, Department of Human Pathology of Adult and Evolutive Age, University Hospital of Messina, Messina, Italy; 5 Infectious and Tropical Disease Unit, Department of Medical and Surgical Sciences, “Magna Graecia” University, Catanzaro, Italy; 6 Clinical Pharmacology and Pharmacovigilance Unit, Department of Science of Health, “Magna Graecia” University, Catanzaro, Italy; 7 Institute of Anesthesia and Intensive Care, Department of Medicine, University of Padua, Padua, Italy

**Keywords:** SARS-CoV-2, COVID-19, Coronavirus, Gallbladder, Cholecystitis, Histopathology, Immunohistochemistry

## Abstract

**Background:**

Since its first documentation, a novel coronavirus (SARS-CoV-2) infection has emerged worldwide, with the consequent declaration of a pandemic disease (COVID-19). Severe forms of acute respiratory failure can develop. In addition, SARS-CoV-2 may affect organs other than the lung, such as the liver, with frequent onset of late cholestasis. We here report the histological findings of a COVID-19 patient, affected by a tardive complication of acute ischemic and gangrenous cholecystitis with a perforated and relaxed gallbladder needing urgent surgery.

**Case presentation:**

A 59-year-old Caucasian male, affected by acute respiratory failure secondary to SARS-CoV-2 infection was admitted to our intensive care unit (ICU). Due to the severity of the disease, invasive mechanical ventilation was instituted and SARS-CoV-2 treatment (azithromycin 250 mg once-daily and hydroxychloroquine 200 mg trice-daily) started. Enoxaparin 8000 IU twice-daily was also administered subcutaneously. At day 8 of ICU admission, the clinical condition improved and patient was extubated. At day 32, patient revealed abdominal pain without signs of peritonism at examination, with increased inflammatory and cholestasis indexes at blood tests. At a first abdominal CT scan, perihepatic effusion and a relaxed gallbladder with dense content were detected. The surgeon decided to wait and see the evolution of clinical conditions. The day after, conditions further worsened and a laparotomic cholecystectomy was performed. A relaxed and perforated ischemic gangrenous gallbladder, with a local tissue inflammation and perihepatic fluid, was intraoperatively met. The gallbladder and a sample of omentum, adherent to the gallbladder, were also sent for histological examination.

Hematoxylin-eosin-stained slides display inflammatory infiltration and endoluminal obliteration of vessels, with wall breakthrough, hemorrhagic infarction, and nerve hypertrophy of the gallbladder.

The mucosa of the gallbladder appears also atrophic. Omentum vessels also appear largely thrombosed. Immunohistochemistry demonstrates an endothelial overexpression of medium-size vessels (anti-CD31), while not in micro-vessels, with a remarkable activity of macrophages (anti-CD68) and T helper lymphocytes (anti-CD4) against gallbladder vessels. All these findings define a histological diagnosis of vasculitis of the gallbladder.

**Conclusions:**

Ischemic gangrenous cholecystitis can be a tardive complication of COVID-19, and it is characterized by a dysregulated host inflammatory response and thrombosis of medium-size vessels.

## Background

In late December 2019, clusters of patients with interstitial pneumonia of unknown cause have been reported by some local health facilities in Wuhan (China). On January 7, the Chinese Center for Disease Control identified a novel coronavirus (SARS-CoV-2) [1], consequently declared a pandemic disease (COVID-19) by the World Health Organization on March 11.

COVID-19 mainly affects male patients (around 60%), with a median age of about 50 years; 40% of patients develops a severe acute respiratory failure (ARF), 5% requiring intensive care [2, 3]. COVID-19 is also characterized by a high intensive care unit (ICU) mortality rate of about 26%, with a death rate higher among older patients [4].

The most common symptoms are fever followed by dry cough, shortness of breath, dyspnea, chest pain, fatigue, and myalgia [5]. Less common symptoms include headache, dizziness, abdominal pain, diarrhea, sputum production, abdominal pain, nausea, and vomiting [5]. Approximately 75% of patients show bilateral pneumonia [6]. Different from other coronavirus infections, COVID-19 has a greater preference for infecting the lower respiratory tract, with severe forms of ARF complicated by shock and acute organ failures [5, 6].

Very recently, a case series have reported that liver injury is a frequent, although transient and non-severe complication of COVID-19. Authors reported that late cholestasis was frequently observed, while synthetic function was preserved. They hypothesized that cholestasis may be associated to several factors, such as inflammation, parenteral nutrition, or drug toxicity [7]. We report, for the first time, the histological findings of a COVID-19 patient, affected by a tardive complication of acute ischemic gangrenous cholecystitis with a perforated and relaxed gallbladder needing urgent surgery.

## Case presentation

On March 24, a 59-year-old Caucasian male (78 kg of actual body weight, 175 cm of height) was admitted to a peripherical hospital for progressive respiratory symptoms. In the 12 days preceding hospital admission, the patient reported fever and sore throat. Due to the medical history, a nasal and pharyngeal swab was performed and SARS-CoV-2 infection confirmed. Interleuchin-6 (IL-6) was 2110 pg/mL (normal range < 7 pg/mL). Intravenous tocilizumab 600 mg was immediately administered. Twelve hours after the first dose, physicians did not observe clinical and/or blood test improvements, so they decided to administer a second dose.

The patient’s condition progressively worsened, and he was transferred to our tertiary-level ICU. At the arrival, the patient was awake and conscious; he was spontaneously breathing through Venturi mask, with a respiratory rate > 30 breaths/min; the oxygen arterial partial pressure (PaO_2_) to inspired fraction (FiO2) ratio (PaO_2_/FiO_2_) was 163 mmHg at the arterial blood gases (ABGs). Hemodynamics were initially stable, with a mean arterial blood pressure (MAP) > 65 mmHg. Sequential Organ Failure Assessment (SOFA) score was 11.

Therefore, continuous positive airway pressure (CPAP) through helmet in combination with prone position was firstly attempted [8]. All routine microbiological cultures (blood, urine, and surveillance for multidrug-resistant bacteria) were also collected. However, due to the lack of ABGs improvement after 2 h and presence of respiratory distress and tachypnea, we decided for immediate intubation and protective invasive mechanical ventilation (iMV), in volume-controlled mode, with continuous infusion of rocuronium 0.6 mg*kg/h [9]. Despite fluid resuscitation, MAP was < 65 mmHg, deeming necessary the administration of norepinephrine at 0.30 mcg/kg/min. After 1 h of iMV, ABGs showed a moderate alteration of oxygenation with a PaO_2_/FiO_2_ of 186 mmHg.

Blood test at admission and along the first 3 weeks of ICU stay are reported in Table 1. A general inflammatory status with an acute injury of the liver and kidney, and an abnormal increase of cardiac and muscular enzymes (in absence of electrocardiogram alterations) were observed. At ICU admission, IL-6 was 1704 pg/mL, despite treatment with tocilizumab. Microbiological cultures resulted positive for *Klebsiella pneumoniae* and *Candida albicans* in rectal and pharyngeal swabs, respectively.

**Table 1:** Blood test along the ICU admission

	Normal range	Day 0	Day 3	Day 6	Day 9	Day 12	Day 15	Day 18	Day 21
White blood cell (*n*/μL)	4.5-11	23.8	21.00	24.00	14.00	12.78	31.05	57.35	16.36
Neutrophils (%)	45-62	85.2	81.9	85.3	79.5	15.3	77.8	86.4	69.1
Lymphocytes (%)	16-33	7	10.7	7.7	10.6	12.8	11.2	7.8	14.8
Platelets (*n* × 10^3^/μL)	150-400	118	193	220	139	136	150	169	163
Procalcitonin (ng/mL)	< 0.2	0.87	0.31	0.28	0.19	0.20	0.27	2.45	1.07
Troponin (ng/L)	< 14	30	20	25.4	48.2	48.1	42.7	94.2	96.6
Myoglobin (ng/mL)	25-72	93	486	266	426	274	173	368	224
CK-Mb (ng/mL)	< 3.61	1.4	1.2	1.2	1.2	2.4	1.2	4.4	5.2
Lactate dehydrogenase (IU/L)	< 600	1560	1160	804	407	620	755	816	696
Creatinine (mg/dL)	0.8-1.2	2.27	2.12	2.29	2.12	2.19	2.66	3.18	3.4
Alanine aminotransferase (IU/L)	≤ 34	146	52	58	38	59	45	43	34
Aspartate aminotransferase (IU/L)	≤ 34	74	78	86	50	49	51	30	28
Total bilirubin (mg/dL)	< 1.40	1.10	0.92	1.14	1	1.43	1.20	0.63	0.42
Conjugated bilirubin (mg/dL)	< 0.40	1.09	0.69	0.66	0.64	0.83	0.63	0.40	0.28

Intravenous empiric antimicrobial therapy was started with meropenem 1 g thrice-daily and linezolid 600 mg twice-daily, in addition to SARS-CoV-2 treatment with azithromycin 250 mg once-daily and hydroxychloroquine 200 mg trice-daily. Enoxaparin 8000 IU twice-daily was administered subcutaneously. Despite it, thrombosis of jugular and femoral veins occurred, without signs of pulmonary embolism along the ICU stay. Prone position was also performed during iMV. Due to worsening of kidney function, continuous renal replacement therapy was performed.

At day 8 of ICU admission, blood test and gas exchange significantly improved and the patient was extubated and weaned off from iMV through helmet non-invasive ventilation (NIV) [10] in a proportional mode to improve patient-ventilator interaction and to increase the rate of success [11–14]. After 48 h, weaning from NIV was performed with high-flow oxygen through nasal cannula, to unload respiratory muscles and provide heated and humidified air-oxygen admixture [15–17].

In the next days, patients continued renal replacement therapy and a low dose of vasoactive agent (norepinephrine < 0.3 mcg/kg/min) due to hypotension. At day 15, WBC count increased with occurrence of fever (> 38.5 °C), due to a catheter-related bloodstream infection due to a methicillin-resistant staphylococcus aureus; antimicrobial therapy was initiated with linezolid 600 mg twice-daily for ten consecutive days.

**Fig. 1:**
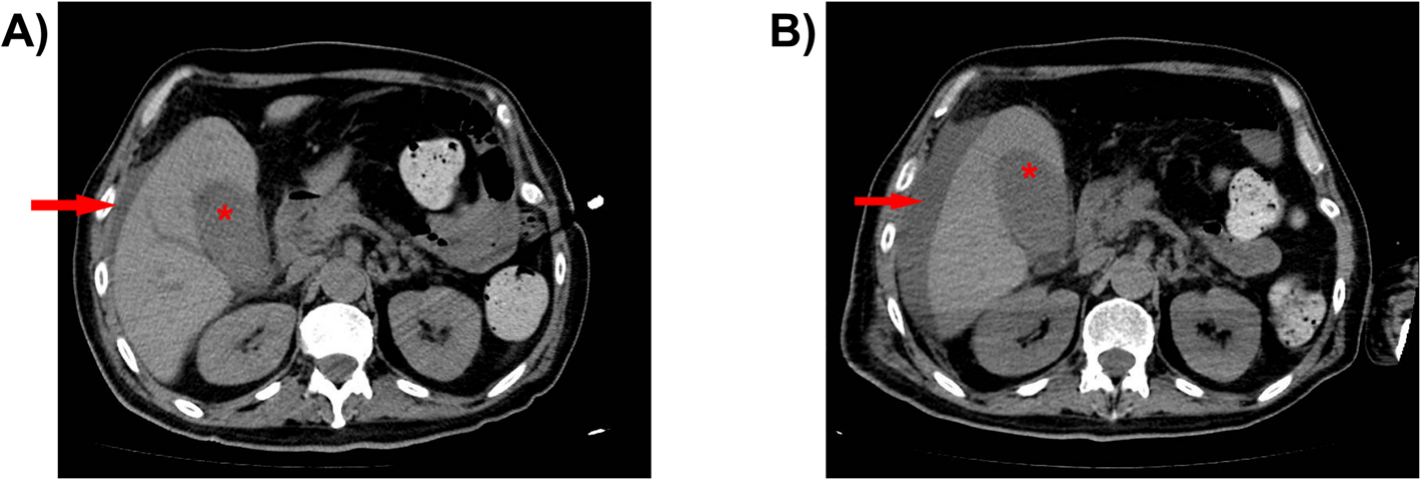
The abdomen CT scan of day 32 (**a**) and day 33 (**b**) show relaxed gallbladder with dense content (asterisks) and perihepatic effusion (arrows), which increased in the second CT scan (**b**)

At day 32, the patient revealed abdominal pain without signs of peritonism at examination. At blood tests, white blood cells (18.94 n/mL), procalcitonin (2.73 ng/mL), and cholestasis indexes rapidly increased. Empiric antibiotic therapy was reinstituted with meropenem 1 g trice-daily and tigecycline 50 mg twice-daily. The abdominal CT scan (Toshiba Aquilon 64 Slices, Toshiba, Tokyo, Japan) detected perihepatic effusion and a relaxed gallbladder with dense content (Fig. 1a). Surgical consultancy was required, and the consultant suggested to wait and see the evolution of clinical conditions. The day after (day 33), blood tests further worsened, as well as symptoms. Based on a second abdominal CT scan showing increased perihepatic effusion (Fig. 1b), surgeons decided for a laparotomic cholecystectomy; laparotomy was preferred over laparoscopy to limit virus spread according to the internal protocol, although not clearly demonstrated by the literature [18]. A relaxed and perforated gallbladder, with a local tissue inflammation and perihepatic fluid, was intraoperatively met. Gallbladder and a sample of omentum, adherent to gallbladder, were also sent for histological examination.

During surgery, swabs for detection of SARS-CoV-2 were performed on perihepatic fluid, bile, and rectum. All swabs resulted negative for SARS-CoV-2 infection. Nasopharyngeal swabs were also negative. The day after surgery, the patient was extubated and he progressively improved till day 44, when he was discharged to a COVID-19 ward.

### Histological and immunohistochemistry methods

The histological diagnosis was made on hematoxylin-eosin-stained slides. A three-layer biotin-avidin-peroxidase system was utilized to appraise endothelial cells, macrophages, and lymphocytes CD4^+^ (helper) [19–21]. Briefly, 4-μm thick serial sections of formalin-fixed and paraffin-embedded samples were deparaffinized. Afterward, sections were microwaved at 500 W for 10 min for antigen retrieval and endogenous peroxidase activity was blocked with 3% hydrogen peroxide solution. Slides were subsequently incubated with (1) monoclonal antibodies anti-CD31 (clone JC70A; DAKO, Glostrup, Denmark), diluted at 1:40 for 30 min at room temperature and pH 8, to identify endothelial cells; (2) monoclonal antibodies anti-CD68 (Clone KP1; DAKO, Glostrup, Denmark), diluted at 1:100 for 1 h at room temperature and pH 8, to identify tissue macrophages; and (3) monoclonal antibodies anti-CD4 (Clone 4B12; DAKO, Glostrup, Denmark) diluted at 1:100 for 20 min at temperature of 97 °C and pH 8, to identify lymphocytes CD4^+^.

The bound antibody was visualized using DAB Chromogen brown secondary antibody (3,3′-diaminobenzidine, DAKO, Glostrup, Denmark). Nuclear counterstaining was performed with Gill’s hematoxylin no. 2 (Polysciences, Warrington, PA, USA).

### Morphometrical assay

Serial sections of each sampled tissue were evaluated through light microscopy integrated with an image analysis system (Olympus, BX53M, Tokyo, Japan). In tissue sections, five most immunostained areas (hot spots) were selected at low (× 2/0.08 NA) magnification; details were then evaluated at high magnification (× 10-40/0.40 NA). In case of cell aggregates, immune cells were identified through immunostaining, presence of perinuclear cytoplasmatic area, and blue staining of the nucleus.

### Histological findings

**Fig. 2:**
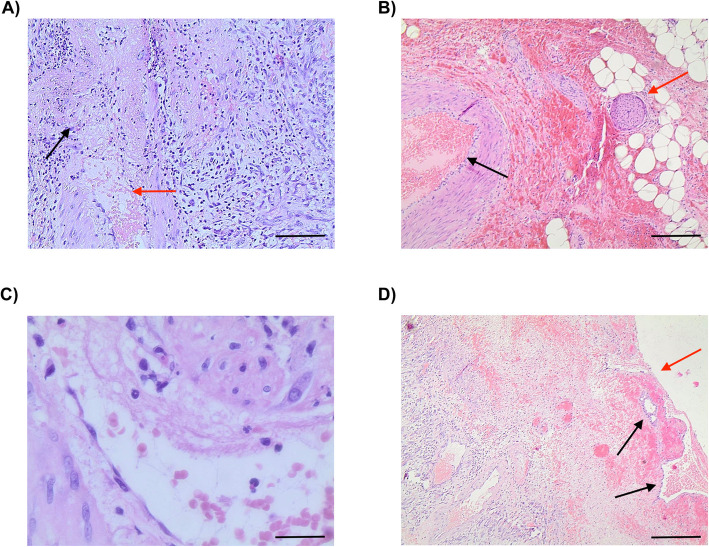
Hematoxylin-eosin-stained sections of the gallbladder. Inflammatory infiltrates diffusely involve medium-size arteries, with obliteration of their lumen. These features indicate vasculitis with thrombosis. **a** Lumen obliterated by inflammatory cells with wall breakthrough is indicated by a red arrow, while normal lumen by a black arrow (magnification × 2/0.08 NA); a further magnification of the tissue is represented in **c** (magnification × 40/0.40 NA). **b** The black arrow indicates an ischemic obliteration, while the red arrow highlights the presence of nerve hypertrophy (magnification × 2/0.08 NA). **d** The gallbladder mucosa appears to be atrophic (red arrow); two glands are indicated by black arrows (magnification × 2/0.08 NA)

As shown in Fig. 2, hematoxylin-eosin-stained slides display the inflammatory infiltration and endoluminal obliteration of vessels, with wall breakthrough, hemorrhagic infarction (Fig. 2a and b, black arrow), and nerve hypertrophy of the gallbladder (Fig. 2b, red arrow).

In Fig. 2c, a detail of Fig. 2a at high (× 40/0.40 NA) magnification highlights inflammatory infiltrates with wall breakthrough. In Fig. 2d, the mucosa of the gallbladder appears also atrophic (red arrow), while glands are few, although normal (black arrows).

**Fig. 3:**
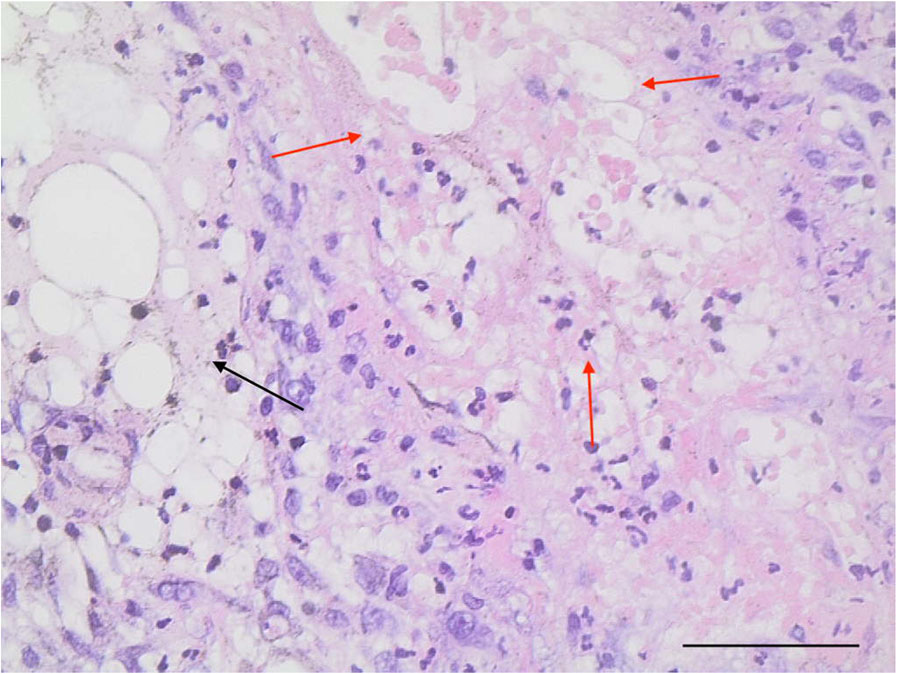
Normal adipose tissue is represented in the omentum (black arrow), while vessels are thrombosed (red arrows) (magnification × 2/0.08 NA)

Figure 3 shows the involvement of the omentum in the disease process; in particular, omentum vessels appear largely thrombosed (red arrows), while adipose tissue being normal (black arrow).

**Fig. 4:**
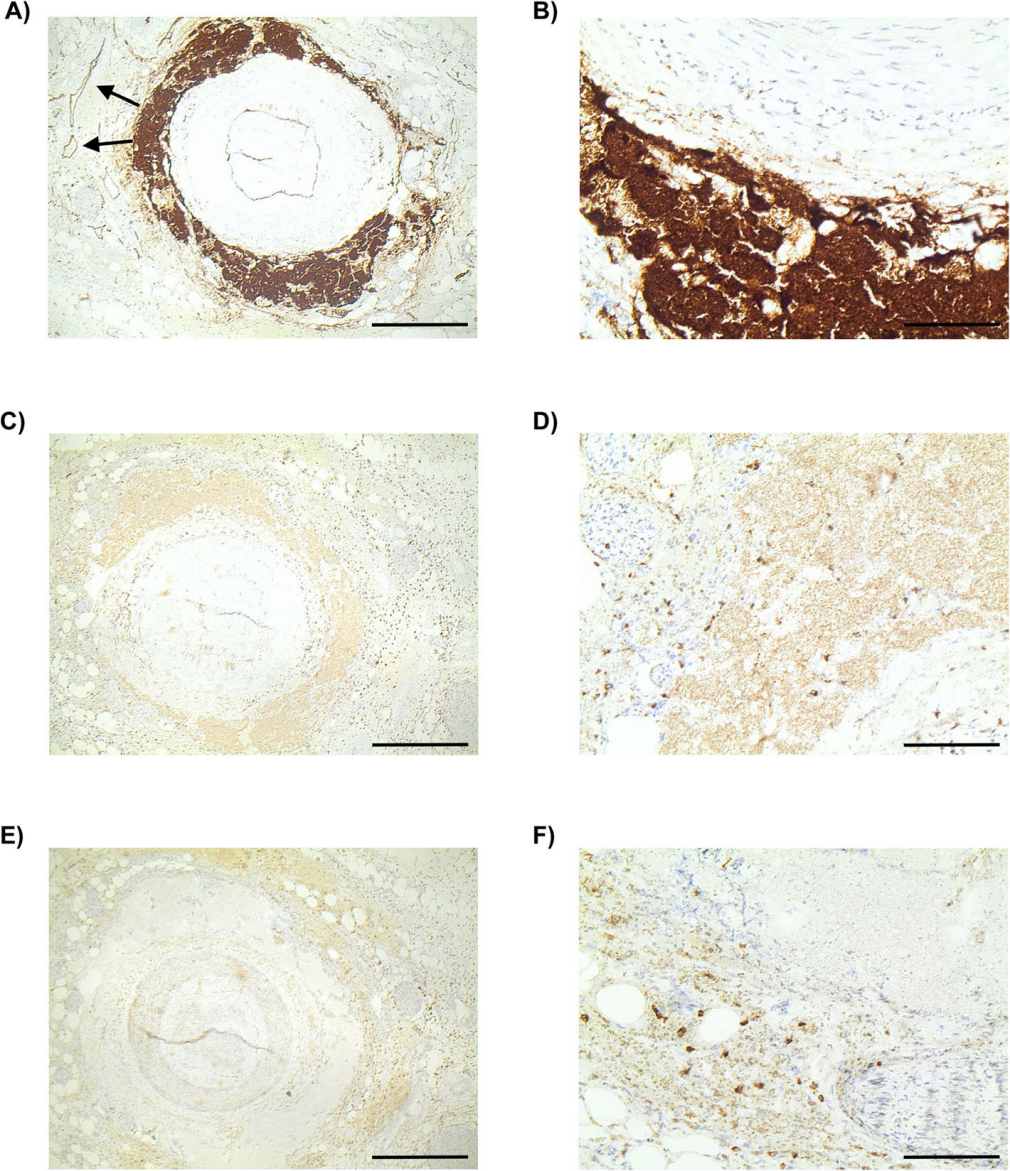
Immunohistochemical images relative to (1) over endothelial cells expression with anti-CD31 antibody (dark brown) in **a** (magnification × 2/0.08 NA) and in **b** (magnification × 10/0.40 NA); (2) tissue macrophages with anti-CD68 antibody (brown) in **c** (magnification × 2/0.08 NA) and in **d** (magnification × 10/0.40 NA); and (3) tissue lymphocytes CD4+ (helper) with anti-CD4 antibody (brown) in **e** (magnification × 2/0.08 NA) and in **f** (magnification × 10/0.40 NA)

Figure 4 shows immunohistochemical findings relative to anti-CD31 (Fig. 4a and b), anti-CD68 (Fig. 4c and d), and anti-CD4 antibodies (Fig. 4e and f) at low and high magnification. Immunohistochemistry demonstrates an endothelial overexpression of medium-size vessels (anti-CD31), while not in micro-vessels, with a remarkable activity of macrophages (anti-CD68) and T helper lymphocytes (anti-CD4) against gallbladder vessels. All these findings define a histological diagnosis of vasculitis of the gallbladder.

## Discussion

To the best of our knowledge, this is the first report of histopathological findings of an acute ischemic gangrenous cholecystitis as a tardive complication in a patient affected by SARS-CoV-2 infection.

SARS-CoV-2 is characterized by the presence of a viral protein Spike (commonly referred to as “S”) that interacts with the angiotensin-converting enzyme-2 receptor (ACE2) in the host [22]. ACE2 is a protein highly expressed in the lungs, intestine, oral mucosa, and liver. The expression of ACE2 in the liver can explain the development of liver injury characterized by a reversible increase in aspartate aminotransferase (AST) and alanine aminotransferase (ALT), which mainly occurs in the first week of the disease [23]. However, it remains under debate if SARS-CoV-2 is directly responsible of the liver injury, or whether the liver damage is secondary to the systemic inflammatory response of COVID-19. In addition, during COVID-19 the development of the liver injury might also due secondary to tissue hypoxia or drug-induced toxicity [23].

In our patient, we report the presence of overexpression of endothelial cells in medium-size vessels (while not in microvessels), vascular lumen obliteration by inflammatory cells with wall breakthrough, thrombosed vessels, and hemorrhagic infarction of the gallbladder. In fact, expression of endothelial cells could trigger a cytokine storm which recruits macrophages and causes inflammatory reactions, similar to those of vasculitis, and the activation of a thrombophilic status [24]. Indeed, SARS-CoV-2 infection upregulates the expression of pro-inflammatory cytokines, such as IL-6 and tumor necrosis factor-alpha (TNF-α). In case of severe inflammatory response, both IL-6 and TNF-α activate the coagulation cascade [25, 26], explaining the presence of thrombosed vessels in the gallbladder and the occurrence of an ischemic gangrenous cholecystitis. A similar pattern of the thrombosed vessel has been found also in the omentum. Of note, diffused thrombosis occurred also in the jugular and femoral veins, despite the prophylactic high dose of enoxaparin.

Interestingly, in a case series of 7 critically ill adults affected by COVID-19 was reported the occurrence of acro-ischemia lesions including finger/toe cyanosis, skin bulla, and dry gangrene [27]. In 4 out of 7 patients, disseminated intravascular coagulation (DIC) was diagnosed [27]. In another report from Wuhan, 71.4% of dead COVID-19 patients showed evidence of overt DIC [28]. The dysregulation of coagulation and the urokinase pathway was already reported by Gralinski et al. during the coronavirus SARS disease that emerged in 2002 and 2003 [29]. More recently, in three patients affected by COVID-19, the presence of antiphospholipid antibodies was reported, which may lead also to thrombotic events [30]. Therefore, it is becoming clearer and clearer that dysregulated coagulation is implicated in complication related to SARS-CoV-2 infection.

However, it remains unclear the absence of SARS-CoV-2 detection in the multiple swabs we performed. In keeping with previous report [31], we could not clearly demonstrate the presence of the SARS-CoV-2 infection through pharyngeal and nasal swabs. In fact, the SARS-CoV-2 swabs may be already negative after 2 weeks, while viral load increases over 2-3 weeks in deeper respiratory secretions (such as sputum and bronchoalveolar fluid), and viral shedding is prolonged in stool [32]. Nevertheless, rectal, perihepatic fluid, and bile swabs were all negative. Of note, for the first time, it has been recently reported the presence of SARS-CoV-2 in the peritoneal fluid, in concomitance with nasal and pharyngeal positive swabs [33]. We cannot therefore exclude that the viral shedding already occurred in our patient, leading to the hypothesis that the inflammatory status might constantly be present, even for a certain period after a complete viral shedding.

## Conclusions

In conclusion, in our patient with a recent SARS-CoV-2 infection, ischemic gangrenous cholecystitis can be a tardive complication of COVID-19, and it is characterized by a dysregulated host inflammatory response and thrombosis of medium-size vessels. Further data are deemed necessary to confirm such observation in other cases.

## Data Availability

All data generated or analyzed during this study are included in this published article.

## Abbreviations

ABGs: Arterial blood gases
ACE-2: Angiotensin converting enzyme 2
ALT: Alanine aminotransferase
ARF: Acute respiratory failure
AST: Aspartate aminotransferase
COVID-19: Coronavirus disease
CPAP: Continuous positive airway pressure
DIC: Disseminated intravascular coagulation
FiO_2_: Inspired oxygen fraction
ICU: Intensive care unit
iMV: Invasive mechanical ventilation
IL-6: Interleuchin-6
MAP: Mean arterial pressure
NIV: Non-invasive ventilation
PaO_2_: Arterial partial pressure of oxygen
PaO_2_/FiO_2_: Arterial partial pressure to inspired fraction of oxygen ratio
SARS-CoV-2: Severe acute respiratory syndrome coronavirus 2
SOFA: Sequential Organ Failure Assessment
TNF-α: Tissue necrosis factor α
WBC: White blood cell
